# From Single Cell to Plants: Mesophyll Protoplasts as a Versatile System for Investigating Plant Cell Reprogramming

**DOI:** 10.3390/ijms21124195

**Published:** 2020-06-12

**Authors:** Taras Pasternak, Kateryna Lystvan, Alexander Betekhtin, Robert Hasterok

**Affiliations:** 1 Institute of Biology II/Molecular Plant Physiology, Centre for BioSystems Analysis, BIOSS Centre for Biological Signalling Studies University of Freiburg, 79104 Freiburg, Germany; 2 Institute of Cell Biology and Genetic Engineering of the National Academy of Sciences of Ukraine, 03143 Kyiv, Ukraine; lystvan@icbge.org.ua; 3 Plant Cytogenetics and Molecular Biology Group, Institute of Biology, Biotechnology and Environmental Protection, Faculty of Natural Sciences, University of Silesia in Katowice, 40-032 Katowice, Poland; alexander.betekhtin@us.edu.pl

**Keywords:** cell cycle, epigenetics, protoplasts, reprogramming, totipotency

## Abstract

Plants are sessile organisms that have a remarkable developmental plasticity, which ensures their optimal adaptation to environmental stresses. Plant cell totipotency is an extreme example of such plasticity, whereby somatic cells have the potential to form plants via direct shoot organogenesis or somatic embryogenesis in response to various exogenous and/or endogenous signals. Protoplasts provide one of the most suitable systems for investigating molecular mechanisms of totipotency, because they are effectively single cell populations. In this review, we consider the current state of knowledge of the mechanisms that induce cell proliferation from individual, differentiated somatic plant cells. We highlight initial explant metabolic status, ploidy level and isolation procedure as determinants of successful cell reprogramming. We also discuss the importance of auxin signalling and its interaction with stress-regulated pathways in governing cell cycle induction and further stages of plant cell totipotency.

## 1. Introduction

Cells of higher plants can retain their regenerative potential when cultured in vitro. Some cell types are able to regenerate organs or even whole plantlets through organogenesis or somatic embryogenesis. Some cell types of monocotyledons at certain developmental stages are able to re-enter the cell cycle, but their regenerative capacity is not stable and is usually lost rapidly after induction of cell division [1]. Even in dicotyledonous plants, the ability of plant regeneration is strictly dependent on the genotype and the age of a cell, i.e., only young explants retain regenerative potential [2,3]. The causes of this phenomenon are still poorly understood and cannot be readily explained genetically because all genotypes in planta have similar meristems and a comparable ability to undergo zygotic embryogenesis. Previously, the changes that occur during cell differentiation were attributed only to cell cycle progression kinetics, which explained differentiation in terms of regulation of cell cycle genes [4,5]. According to this view, cell division (proliferation) is the most likely driver of cell reprogramming. However, many phenomena cannot be explained by genetics and gene expression profiles alone. In their recent review, Velappan, et al. [6] discussed the differences between dormancy and differentiation and concluded that cell differentiation in plants is not just accompanied but is essentially regulated by changes in the chromatin structure. Recent investigations have indicated the key role of epigenetics, i.e., alterations in the chromatin structure and accessibility, in regulating both cell differentiation and de-differentiation [7,8,9].

The phenomenon of plant cell reprogramming and its mechanisms has been investigated using different systems that are based mainly on multicellular explants originating from leaves or roots [10,11]. Such explants contain different cell types with different epigenetic and metabolic profiles. These cells can respond in different ways to de-differentiation stimuli, which complicates the interpretation of data from such regeneration systems. This emphasises the importance of studying and using single cell systems.

## 2. Cell Differentiation and De-Differentiation in Planta

Cell differentiation or specification in planta occurs during embryogenesis and post-embryogenic development, but there are few reports about cell programming during the latter [12,13]. Among the various cell specifications, one of the most important and well-known is the formation of two stem cell poles in the shoot apical meristem (SAM) and the root apical meristem (RAM), which are regulated by the activity of the *WUSCHEL* (*WUS*) and *WUSCHEL-like homeobox* (*WOX*) genes, respectively [14,15]. Thereafter, embryos enter the seed’s dormancy stage, which hampers investigation of the conversion from cell specification to cell differentiation. Therefore, investigating this conversion is possible only in artificial systems because immature embryos (10–14 days after pollination) skip the dormancy step and are directly converted into plants. The formation of flowers and its biogenesis is another example of cell reprogramming. This process is accompanied by changes in the epigenetics of a cell and, correspondingly, its fate [16]. The process of differentiation, with a focus on chromatin structure, is well described for shoot development in *Arabidopsis thaliana* (Arabidopsis) [17]. Briefly, cells that originate from the SAM pass through three steps of differentiation: (i) rapid cell proliferation in the young leaves, (ii) endocycles in the expanding leaves, and lastly, (iii) terminal differentiation in old leaves. Similar steps have been observed in roots during cell differentiation, which have a clear dependence on the differentiation and age of the cell. Chromatin modifications play a key role in the acquisition and maintenance of cell fate during all stages of cell differentiation [18]. The speed of chromatin remodelling and the resulting cell status in each plant "zone" differ markedly between species. All of the aforementioned three stages are regulated by alterations in epigenetic marks, which modify chromatin structure: a gradual histone de-acetylation with a concomitant methylation of histones at certain positions, as well as DNA methylation [19]. The prominent role of epigenetics in cellular differentiation was also pointed out by Mohn and Schübeler [20]. Cell de-differentiation, i.e., the conversion of differentiated cells into totipotent (stem-like) cells in planta is a process that reverses cell differentiation and is likely to involve the same steps in reverse.

## 3. Experimental Systems for Exploiting Totipotency

Several experimental systems have been developed to exploit plant cell totipotency. In dicotyledonous plants, “leaf disks” have been used widely since 1985 [21]. This experimental approach is often integral to *Agrobacterium*-mediated transformation in a number of dicotyledonous species [22]. In recent years, the molecular mechanism of plant regeneration from the leaf tissue has been investigated in detail in several plant species [3,23], focusing mainly on the epigenetic status of cells during reprogramming. The recent study of Sun, et al. [24] convincingly demonstrates the strict dependence of totipotency competence on DNA methylation status and leaf age.

Callus formation is another example of somatic cell reprogramming, i.e., the activation of divisions in differentiated cells towards potential totipotency. Recently, the epigenetic and molecular mechanisms of this transition have become clearer (for review, see [23,25]) and the importance of histone acetylation in Arabidopsis root cell reprogramming has received most attention [26]. However, there are two major drawbacks in using callus to investigate cell reprogramming: (i) the explants are not uniform and (ii) not all callus cells can convert to the totipotent stage [27]. The main problem in this case is that different cell types respond differently to in vitro culture conditions, sometimes even in the opposite direction intended. As a classic example, in Arabidopsis root segments cultured on a callus-induction medium, only the phloem-pole pericycle cells actively divided, and induced callus formation with specific epigenetic changes; while, at the same time, the cortex/epidermis cells underwent apoptosis and had an epigenetic landscape that was significantly different from that of the pericycle cells. The key role of histone and DNA modifications has also been shown in the regulation of this process [28].

Moreover, these systems cannot be used in most monocotyledonous plants because leaf tissue is refractory to re-entering the cell cycle [1,29]. For example, leaf tissue in a small number of barley genotypes is able to re-enter cell divisions in the region close to meristem cells [30]. Immature embryos and inflorescences have been widely used to induce embryogenic callus in monocots [31,32,33]. The biological processes underpinning these differences, as well as the epigenetic mechanisms of totipotency in monocotyledonous plants including cereals, have not been well studied.

In multicellular systems, the effects of neighbouring cells and internal hormonal signalling on cell reprogramming cannot generally be isolated. Therefore, these systems cannot be used effectively to study the effects of hormones, when, for example, certain cells in the leaf continuously synthesise auxins. Moreover, tracking individual cells is impossible in a multicellular system. In order to avoid such complexity and to simplify the approach, a homogeneous cell population without connections to neighbouring cells is required. One possibility is to use the single-cell system of isolated microspores [34]. The main advantage of this approach is that microspores are relatively highly homogeneous and represent a “natural single cell system”. After meiosis, microspores can be considered to be partially differentiated cells and their transition to embryogenic cell divisions can be considered an example of cellular reprogramming. The classical experiment of tobacco microspore reprogramming from the gametophyte to sporophyte pathway indicates that stress is a necessary factor for this switch [35]. Recently, it has also been demonstrated that auxin metabolism is the main target of such stresses [36].

This system is very useful for generating haploid and doubled haploid plants for breeding [34], but from a practical point of view microspores have serious limitations for studying cell reprogramming: (i) a long time is needed to obtain sufficient starting material. For example, even the rapid life cycle of Arabidopsis takes at least 40 days to produce microspores, and in other species, this could be much longer; (ii) very precisely controlled greenhouse conditions are required at different stages of plant development [37,38]; (iii) after meiosis, microspores undergo differentiation very rapidly, which leads to a low frequency of cells undergoing reprogramming; and (iv) only a limited number of genotypes have a relatively high frequency of embryogenesis [38,39].

An alternative, more amenable system involves the use of isolated protoplasts which overcomes most of the limitations of the microspore system described above [40]. Protoplasts are a particularly useful material for studying cell reprogramming because they comprise a relatively homogeneous population that lacks cell-to-cell communications and can be easily isolated and cultivated under different conditions [41]. Their main advantages are: (i) they can be isolated even from young seedlings, i.e., five to six days after the seeds have germinated, thereby saving much time; (ii) the amount of protoplasts isolated is practically unlimited; for example, 100 mg of six- to seven-day-old Arabidopsis seedling material contains up to three million cells, which can provide at least 200,000 viable protoplasts and (iii) the biological age and epigenetic status of the starting material can be experimentally selected, allowing the process of cell de-differentiation to be studied in cells of different status [42].

## 4. Using Protoplasts to Study Cell De-Differentiation

The term protoplast originates from the ancient Greek word prōtóplastos, which means “first-formed”, and was proposed by Hanstein in 1880 to refer initially to a cell without a cell wall. One of the first successful protoplast isolations using enzymatic digestion was performed in 1965 from the parenchymatous placental tissue of immature tomato fruit by Gregory and Cocking [43]. Later, it was demonstrated that protoplasts can re-engage in cell division and form a callus, opening the possibility of plant regeneration (for review, see [44]). One of the first demonstrations of somatic embryogenesis was reported from carrot protoplasts in 1976 [45]. Thereafter, direct plant regeneration from protoplasts of mesophyll cells was achieved for a number of dicotyledonous species (for review, see [46]). The majority of investigations have been devoted to analysing gene expression during cell reprogramming, but detailed molecular genetics and epigenetic mechanisms of totipotency in angiosperms remain elusive.

### 4.1. Protoplast Sources

A reliable source of quality protoplasts is necessary for studying cell reprogramming. There are two main criteria for selecting the tissue sources of protoplasts: (i) the type of organs and (ii) the type of the cells comprising the organs, i.e., cell competence. Protoplasts can be isolated from the leaves (mesophyll protoplasts), roots (root protoplasts) and callus (callus protoplasts). Protoplasts isolated from different organs have different biological profiles and therefore require different culture conditions. One of the most important factors is the status of the donor plant cells: differentiated with a low level of chromatin accessibility, e.g., mesophyll cells, or non-differentiated with a potentially active cell cycle and a high level of chromatin accessibility, e.g., callus and to some extent roots. Potential protoplast sources and their possible applications are listed in Table 1.

#### 4.1.1. Shoot-Derived Protoplasts

This type of protoplast can be categorised into several subtypes, including cotyledon, hypocotyl and mesophyll protoplasts. Cotyledon protoplasts can be isolated from relatively young cotyledons before their cells undergo a terminal differentiation. The main advantage of this protoplast type is the relative uniformity of the starting material because all the cotyledons are the same age and have the potential to reach various levels of differentiation. The ability to determine to what extent the differentiation is reversible is a crucial point in an investigation of cell reprogramming. However, one has to consider the irregular ploidy of cells after the endocycles, and therefore, cotyledons can only be used as a protoplast source before entering the endocycles. The rapid process of differentiation in cotyledons is linked with the function of these organs in planta: the large cells with enhanced macromolecular production may require an increase in nuclear DNA contents, which fits well with cotyledon function as the carbohydrate source during early stages of seedling development. Later, the endopolypoid cells expand significantly and undergo terminal differentiation with a high level of chromatin condensation. Endopolyploidy in Arabidopsis may be directly linked with regulating cell size as a possible adaptation mechanism for growth of its relatively small cells [48]. However, it is technically challenging to isolate cotyledon protoplasts from Arabidopsis because the cotyledons are minute and difficult to separate from the very rapid formation of young leaves.

Hypocotyl protoplasts can be isolated from dark-grown seedlings and have similar advantages as cotyledon protoplasts, i.e., they provide a rather homogeneous and synchronised cell population [49]. However, the main disadvantage of both cotyledon and hypocotyl protoplast sources, especially from dark-grown hypocotyls, is the rapidly increasing cell ploidy level. For example, after five days, dark-grown Arabidopsis hypocotyls can have up to 30% of 16C cells [50]. A further disadvantage of this protoplast system is the large amount of seeds that are required and the time-consuming seed plating.

Because of the disadvantages detailed above, mesophyll protoplasts are the most commonly used among shoot-derived protoplasts. They can be isolated from differentiated mesophyll cells of different biological ages as well as from those at different stages [51,52]. The main advantage of mesophyll protoplasts that are isolated from dicotyledonous species, with the exception of in vitro grown Arabidopsis, is the possibility to obtain large amounts of relatively homogeneous cells. The developmental age of the leaves can also be determined [53], which is a critical step for explanting [24]. In dicotyledonous species, selecting the leaves for protoplast isolation is governed by the aim of the experiment, as leaves of different biological ages have differing capacities for cell de-differentiation. The ideal approach is to use only one fully expanded leaf as the protoplast source and to avoid cutting the main vein. Alternatively, two to three leaves that have the same position on the plant are also suitable. This is quite easy to achieve for dicots in which the mesophyll cells in a fully expanded leaf are of a similar age. By contrast, a gradient of differentiation is present in monocots due to their leaf growth, which starts from their base [54]. Mesophyll cells in the leaves of the grasses originate from meristematic cells, which are localised proximally to the meristem and have a rapid exit from the cell cycle. Therefore, only this fraction among isolated cells is capable of cell reprogramming. To date, no successful plant regeneration has been reported from monocotyledon leaf protoplasts.

Another source of shoot-derived protoplasts are guard cells [55], which are considerably more competent than mesophyll cells because of their higher chromatin accessibility and the absence of endocycles, which lead to a more regular chromatin organisation [56]. However, isolating guard cells is considerably more complicated technically than isolating protoplasts from other source cells.

#### 4.1.2. Root Protoplasts

The roots are another option for obtaining a population of isolated cells. Several protocols of root protoplast isolation are available for various species, e.g., various legumes [57,58,59], brassicas [60], *Lycopersicum esculentum* [61], *Quercus rubra* [62] and *Pinus pinaster* [63]. However, protoplast isolation from roots presents a significant technical challenge and does not ensure a homogeneous cell population. The different root zones require different enzyme combinations and different osmotic pressures [64]. This means that the digestion of a whole intact root produces a quite heterogeneous population of different cell types, which prevents a quantitative analysis of the process of cell development.

In conclusion, root protoplasts can be used for biotechnological applications such as fusing or transforming protoplasts but are not optimal for a systematic analysis of cell reprogramming due to their cell heterogeneity. However, the root protoplasts of *Medicago sativa* and some other members of the Fabaceae can be used as efficient models for analysing cell reprogramming during nodule formation [57]. The root protoplasts that are obtained from some monocots and dicots can be useful for patch-clump studies [65].

#### 4.1.3. Callus Protoplasts

A callus comprises disorganised cell masses that are formed in response to hormone treatment and represents a portion of rapidly dividing cells [23]. An embryogenic callus that originates from these structures as immature embryos/inflorescences provides a homogeneous population of relatively non-differentiated cells. However, callus-originated protoplasts of monocotyledons can serve as a tool for studying the induction of cell totipotency. Although this type of protoplast is widely used to study grasses, in particular cereals, for biotechnological applications [66] it is not suitable for investigating the cell de-differentiation mechanisms because the cell cycles of the initial cells have already been activated.

### 4.2. Mesophyll Protoplasts to Study Cell De-Differentiation

Since mesophyll cells provide the most suitable and most popular starting material, we focus here on protoplasts derived from this tissue and describe all of the de-differentiation steps from the differentiated leaf cells to the totipotent cells and somatic embryos. Plant quality and isolation procedure determine the quality of isolated protoplasts, so we focus on these aspects below.

#### 4.2.1. The Role of Optimal Nutrition in Culture Media for Donor Plant Quality and Protoplast Reprogramming

While the growth of donor plants does not need external plant growth regulator (PGR) supplementation, it does require proper internal hormonal balance, which is influenced by nutrient balance [67]. This is particularly important because particular nutrition can prevent a rapid endocycle and cell differentiation. It has been shown that the growth medium for donor plants of Arabidopsis is of significant importance for protoplast culture [68]. In a growth medium, all of the components serve as either a primary building material (N, P) or, as in the case of many micronutrients, contribute to this or other metabolic pathways. The most commonly used growth media often do not accommodate crucial nutrient functions in hormonal signalling because their components have been designed for rapid cell differentiation in the presence of certain phytohormone combinations. The optimal medium for plant growth should prevent nutritional stress, which, in turn, leads to a slowdown in the differentiation gradients in leaf cells and extends the competence window.

#### 4.2.2. Competence Window for Leaf Protoplasts

The concept that cell reprogramming depends on the cell developmental stage first came from an investigation of plant regeneration from the leaf tissue of barley. In this system, the cells undergo very rapid differentiation and only the segments close to the meristem are able to re-enter the cell division cycle [30]. Similarly, immature embryos of wheat also have a very strict competence window [69,70], which occurs when the scutellar tissue (the source of the embryogenic callus) remains active and visually appears to be semi-transparent. This is logical because the process of tissue development from initial cells undergoes several steps but only the early ones are reversible. This is true not only for monocotyledons, but also for dicotyledonous species in which the ability of protoplast regeneration is linked with the biological age of the explants [24]. There are two main reasons for this: (i) chromatin condensation, which can be reversible only under certain conditions and (ii) an irregular ploidy level in differentiated cells, which is irreversible. Therefore, determining the ploidy level and chromatin accessibility in isolated protoplasts is necessary before their culture.

#### 4.2.3. The Protoplast Isolation Step as the Key for Reprogramming

Isolating cells from their native tissue and organ environment can potentially induce apoptosis [71]. This means that the procedure of protoplast isolation is the most important step for ensuring that optimal starting material is obtained. During this procedure, three criteria must be adhered to: (i) the homogeneity of the starting material (only organs of the same biological age can be used, i.e., a single leaf or only the cotyledons); (ii) regular ploidy level of isolated protoplasts. Therefore, ploidy level should be determined using flow cytometry; (iii) the damaging effect of the isolation procedure must be minimised by a gentle cutting, using cellulolytic enzymes, limited centrifugation steps, etc. All of these precautions are particularly important for mesophyll cells, which, once they exit the cell cycle, have condensed chromatin [17], a low level of the “cytoplasmic” antioxidant system and are starting the apoptotic pathway, and whose only function is to supply carbohydrates to developing tissue. The isolation procedure can also induce further chromatin condensation and the apoptotic pathway [72]. This condensation is accompanied by a reduction in the scavenging capacity of reactive oxygen species (ROS) [73], which leads to an increased ROS accumulation [74,75]. Recently, the presence of chromatin condensation has also been demonstrated during protoplast isolation followed by subsequent cultivation in a buffer without PGRs [76].

There are several options for reducing the negative aspects of the protoplast isolation procedure. For example, almost all of the commercially available cellulolytic enzymes are rather crude extracts that contain different proteases/nucleases. Therefore, their removal is crucial for preventing isolated cell degradation, which in turn improves protoplast quality. This can be done either by incubating a crude enzyme solution at 55 °C for 10 min [42] or by decreasing pH to 3.5 for a short time. The ionic composition of a digestion solution is another important consideration: for example, adding certain ions (cell and protoplast washing solution) [77] or antioxidants, such as ascorbic acid, to the enzyme mixture has a significant positive effect on the quality of isolated protoplasts. A good example of such a strategy is the buffer composition for preventing cytosolic acidification that was recently proposed for Arabidopsis [78].

#### 4.2.4. Stages of Mesophyll Protoplast Reprogramming and Accompanying Changes in Their Epigenetic and Physiological Profiles

**Figure 1:**
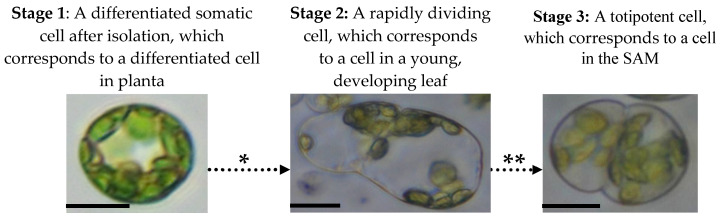
Mesophyll protoplast reprogramming to totipotency. There are three stages that mesophyll protoplasts follow during the activation of their cell division and transition to totipotency which are accompanied by various epigenetic, physiological and molecular processes. The following events occur during mesophyll protoplast reprogramming and cause dynamic changes in chromatin accessibility, hormonal responses and reactivation of the cell cycle: * Chromatin relaxation caused by specific histone and DNA chemical modifications, hormonal/ROS signalling (activation of auxin response and ROS generation/scavenging) and changes in the cell cycle gene expression. ** High-auxin environment leads to protein storage vacuole transition, histone hyperacetylation and cell cycle extension. Photomicrographs show protoplasts of *Medicago sativa*. All bars: 10 µm.

Based on the stages of cell differentiation from the SAM to the mature leaf, we can distinguish three stages in the de-differentiation of the mesophyll protoplast [79,80], which are in reverse to the differentiation stages and which include the induction of the reprogramming process, the epigenetic remodelling of the chromatin and the induction of totipotency (Figure 1).

Changes in the epigenetic chromatin status of differentiated cells is the first step in their conversion to the proliferation pathway [80,81]. There is a dearth of comprehensive studies on the epigenetic changes during mesophyll protoplast cultivation to date. However, according to data that are available for several dicotyledonous species, it is clear that reactivation of the cell cycle is accompanied by increased chromatin relaxation, decreased DNA methylation and changes in histone structure (Table 2).

Besides these epigenetic changes, mesophyll protoplast reprogramming is accompanied by significant modifications in cell structure and physiology including changes in various aspects of its ultrastructure, cytoskeleton and ROS-level vacuolar function.

The first analyses of mesophyll protoplast physiology were performed in the 1970s. These early investigations were reviewed in detail by Galun [86]. Among the physiological parameters that were analysed, oxidative stress responses and changes in the cell ultrastructure were found to be the main hallmarks associated with cell reprogramming. Differentiated mesophyll cells contain a large central lytic vacuole that is characterised by low pH. This vacuole governs the distribution of cytoplasm and organelles to the cell periphery and prevents cell proliferation. During the cultivation period, the vacuole becomes more alkaline [87] and numerous transvacuolar strands arise [88]. Finally, the vacuole divides into several smaller vacuoles, which become protein storage sites during the pro-embryogenic cell divisions [73,83]. The structure of the cytoplasm also changes significantly during the conversion of mesophyll cells into proliferating cells, which is accompanied by changes in the ion composition and total soluble protein profiles. Up to 70% of the soluble protein fraction in mesophyll cells is RuBisCO (~70 kDa), while in proliferating cells, the amount of this enzyme contributes only 10% along with an increasing amount of cytoplasmic proteins.

ROS/redox balance is another key parameter in the cell reprogramming process. Several studies have suggested that the differences in the ROS level between regenerating and non-regenerating protoplasts are the main causes of cell recalcitrance [89,90,91]. Significant changes in ROS generation and scavenging, the antioxidant level, cell structure and vacuolar pH have also been reported [80,87].

### 4.3. Stimuli of Protoplast De-Differentiation: Hormones, Stress and Nutrition

Hormones are a key signal for stimulating cell reprogramming. Among the hormones, auxins are not only required for cell cycle activation [92] and essential for the induction of chromatin relaxation and DNA replication [80] but are also indispensable for somatic embryogenesis in general [93]. Cytokinins are key hormones that are involved in the process of cytokinesis. The complex interaction between these two hormone groups occurs during somatic embryogenesis [94]. Other hormones or PGRs do not seem to be so critical for cell reprogramming but may act by modulating the effects of auxins. For example, the application of brassinolides or salicylic acid can upregulate the auxin signalling in Arabidopsis mesophyll protoplasts. In their study of the *nonphototropic hypocotyl4-1* mutant which is null for the AUXIN RESPONSE FACTOR7 (ARF7) transcriptional activator, Wang, et al. [95] clearly demonstrated the reduced expression of integrated auxin-responsive reporter genes and endogenous genes in Arabidopsis leaf mesophyll protoplasts. Since the mutants of other ARFs did not show any altered expression in reporter or endogenous auxin response genes, it was likely that ARF7 played a major role in regulating auxin effects in leaf mesophyll cells. It further points out that while interactions between hormones and/or PGRs have been investigated in detail at the whole plant level, similar analyses for protoplasts are still lacking and are noteworthy subjects for future investigations.

Stress in combination with auxin is another key factor that is responsible for executing the cell totipotency programme. However, one should distinguish between a stress in response to stress-induced agents, and a combination of stress and hormonal signalling. For example, it has been shown that the application of auxins in combination with stress-inducing agents are required for successful cell reprogramming in *M. sativa* and Arabidopsis and do not lead to actual oxidative stress as determined by H_2_O_2_ level [73,96]. On the other hand, the inhibition of ROS generation and increasing ROS scavenging halt protoplast reprogramming. Low molecular weight antioxidants such as ascorbate and glutathione are considered to be ROS scavengers. Interestingly, while ascorbic acid acts as the main ROS scavenger by inhibiting cell proliferation in both *M. sativa* and *N. tabacum*, glutathione seems to have an opposite effect [81,97]. Nitric oxide seems to have a similar effect [98], which in turn leads to changes in the chromatin architecture [99].

### 4.4. Types of Cell De-Differentiation

De-differentiated cells are different from each other. There are two types of cells in planta: the slow proliferating cells in the SAM and RAM stem cell niches that have an unspecified fate, and the rapidly proliferating ones in the developing organs after cell fate has already been established [19]. Rapidly proliferating cells in roots constitute a population after cell fate has already been determined and therefore cannot give rise to all of the cell types [100]. Only a small portion of stem cells in the SAM and RAM in planta can be considered to be totipotent and these cells are characterised by specific features such as small nuclei, hyperacetylated histones, an extended duration of G1 phase and the presence of protein storage vacuoles. In the majority of cases of reprogramming mesophyll protoplasts, the rapidly proliferating cells can only form a callus, i.e., not totipotent cells. However, it is possible to convert them into SAM-like cells that are able to develop directly into somatic embryos and shoots. From this point of view, the second step in cell de-differentiation is the “creation” of totipotent cells that are capable of being converted to shoots through somatic embryogenesis or organogenesis [101].

### 4.5. Induction of Totipotent Stem Cells from Mesophyll Protoplasts

Only a few cells in whole plants have the features of stem-like cells. Their low abundance makes it difficult to study molecular features of these cells, but culturing protoplasts can circumvent this problem. However, it should be borne in mind that stem-like cells in planta exist in a specific local environment, conditions that need to be reproduced as closely as possible in mesophyll protoplast cultures. By using this approach, it is possible to induce totipotent cells in vitro that have the features of stem-like cells and thus have the potential to generate all cell types. This enables investigation using the standard molecular biology methods of potentially all of the factors that are responsible for stem cell induction in a similar manner to the study of *Physcomitrella patens* [102]. For example, using this approach, Sakakibara, et al. [103] showed differences in gene expression in this model moss and the key role of WOX genes, which serve as epigenetic regulators. In vascular plants, a similar system has been described for *M. sativa* mesophyll protoplasts in which after the application of stress-inducing factors, somatic cells were converted into totipotent cells that had typical stem cell features [73,81,87]. The physiological and genetic mechanisms of this transition include changes in hormonal signalling, cell cycle duration, cell morphology, ROS scavenging activities, content of ascorbate/glutathione, chromatin organisation and gene expression [73,104].

## 5. Conclusions

Leaves are a highly abundant and accessible tissue from which protoplasts can be easily isolated. Therefore, mesophyll protoplasts from dicotyledonous species can serve as a useful model for studying the conversion of differentiated somatic cells into proliferating or totipotent cells. The main advantages of protoplasts are that they can be considered to be “zero-cells” that lack connections with neighbouring cells and therefore lack any external signalling other than those that are applied exogenously. In other words, if cells are the “bricks” that form the plant body, protoplasts are the “clay” for these “bricks” and manipulating this “clay” in different ways enables the “bricks” with the desired characteristics to be obtained. By reproducing microenvironments such as that of stem cells, for example, protoplast development can be guided in various directions and can provide a unique possibility to observe the genuine effects of different development-related factors and phenomena.

Moreover, protoplasts represent a highly homogeneous cell population that is tractable for modern molecular biology and epigenetic methods. The ease of selecting starting material at different biological ages provides opportunities to study the cell de-differentiation process that occurs in cells that have diverse differentiation levels. Using protoplasts from different plant species and sources, enables comparative approaches and enhances the diversity of plant research. However, despite the numerous advantages of using mesophyll protoplasts to gain a better understanding of the systems biology of plant cell reprogramming, the use of systems to date is quite limited. Thus, more research is required to explore and exploit all of the possibilities that are offered by a system that is based on protoplasts.

## Figures and Tables

**Table 1:** Comparison of different angiosperm protoplast sources to study cell reprogramming (summarised from [46,47]).

	Leaf	Hypocotyl/Cotyledon	Root	Callus
Homogeneity	yes	yes	no	yes
Reprogramming from differentiated to proliferating cells	yes	yes	no	no
Potential for totipotency	high for dicots, limited for monocots	high for young explants	high for dicots, limited for monocots	high for dicots and monocots

**Table 2:** Studies on the epigenetic status of mesophyll protoplasts during in vitro culture.

Species	Approach	Process	References
*Nicotiana tabacum*	fluorescence-activated cell sorter (FACS); gel electrophoresis of DNA after micrococcal nuclease (MNase) digestion	chromatin condensation/decondensation	[72]
*Cucumis sativus*	FACS; fluorescence in situ hybridisation	chromocentre and repeat reassembly	[82]
*Medicago sativa*	flow cytometry; nucleus morphology	chromatin relaxation; DNA stainability	[51,80,83]
*Nicotiana tabacum*	nucleus morphology; gene expression	histone H3 modifications; redistribution of HP1; activation of the E2F transcription factor genes	[84]
*Brassica oleracea*; *Cucumis sativus*	quantification of methylated and hydroxymethylated DNA	temporal changes in the amount of 5-mC and 5-hmC	[85]
